# A detachable mobile and adjustable telemetry system

**DOI:** 10.1002/ece3.591

**Published:** 2013-05-18

**Authors:** Tommy S Parker, William E Persons, Joseph G Bradley, Margaret Gregg, Shinelle K Gonzales, Jesse S Helton

**Affiliations:** Urban Wildlife Research Lab, Department of Biology, University of Louisville332 Life Sciences Bldg, Louisville, Kentucky, 40292

**Keywords:** Error distances, error ellipses, mobile telemetry

## Abstract

Many traditional mobile telemetry systems require permanently mounting a rod through the cabin of a vehicle to serve as the mast for a directional antenna. In this article we present an alternative to this configuration by providing a platform that can be placed atop the vehicle in which the antenna mast can be mounted and controlled from the cabin of the vehicle. Thereby making this design a viable option for researchers who share vehicles with others that may not approve of permanent vehicle modifications such as placing a hole in the roof of the vehicle as required by traditional mobile configurations. We tested the precision and accuracy of detachable mobile and adjustable telemetry system (DMATS) in an urban park with varying terrain, tree stands, overhead wires, and other structures that can contribute to signal deflection. We placed three radiocollars 50 m apart and 1.2 m above the ground then established three testing stations ∼280 m from the location of the radiocollars. The DMATS platform required 12 h for completion and cost $1059 USD. Four technicians were randomly assigned radio collars to triangulate using DMATS and a handheld telemetry system. We used a one-way analysis of variance (ANOVA) with a Scheffe post hoc test to compare error ellipses between azimuths taken using DMATS and the hand held system. Average error ellipses for all testers was 1.96 ± 1.22 ha. No significant differences were found between error ellipses of testers (*P* = 0.292). Our design, the DMATS, does not require any vehicle modification; thereby, making this a viable option for researchers sharing vehicles with others that may not approve of permanent vehicle alterations.

## Introduction

Radio telemetry allows the user to track radiocollared animals over large geographic areas in relatively short periods of time (White and Garrott 1990; McClennen et al. 2001). For researchers studying the resource use of wildlife, this technique provides a method to locate animals and assess habitat selection choices made without visually locating the animal (McClennen et al. 2001). However, many of the traditional mobile telemetry systems require a hole to be cut into the roof of the vehicle and the permanent mounting of a rod through the cabin of the vehicle. This rod operates as the mast for one or more directional antenna that receives a very high frequency signal from some type of broadcasting device—namely a radio tag, collar, or implanted device (Brinkman et al. 2002; Cox et al. 2002; Gilsdorf et al. 2008).

The mobile telemetry system developed in the Urban Wildlife Research Lab (UWRL) is detachable, adjustable, and requires no vehicle modification. Thereby making this design a viable option for researchers that share vehicles with others that may not approve of permanent vehicle modifications such as placing a hole in the roof of the vehicle as required by traditional mobile configurations. In addition to the ability to attach and detach the system from a vehicle, the antenna height can be adjusted to provide height options that may enhance the reception of various transmitter frequencies. Furthermore, the height adjustment enables researchers to transport and if necessary, operate the system at minimal height in order to minimize the risk of damage caused by tree branches, overpasses, and/or wires. However, if necessary, the antenna can be raised to a maximum height of 3.7 m.

We are suggesting the detachable mobile and adjustable telemetry system (DMATS) as an alternative to traditional telemetry designs. The materials to build DMATS can be purchased at a local hardware store and constructed using basic tools. The design of DMATS allows additional equipment, such as lights or a predator call, to be added without interfering with the systems primary function of telemetry.

In this article, we present an alternative to traditional mobile telemetry configurations by providing a platform that can be placed atop the vehicle in which the antenna mast can be mounted. Thereby eliminating the need to permanently alter the vehicle in order to provide a hole for the antenna mast. In addition to providing the design for the building of DMATS, we also evaluate the accuracy and precision of the system. To evaluate the accuracy and precision of DMATS we ask the question, “Are azimuths taken from DMATS as accurate as those taken from a hand-held telemetry system?” The goal of this article is to provide a tool that will offer researchers the ability to conduct telemetry studies without the permanence of a traditional mobile telemetry system. Our specific objectives were (1) to introduce a comprehensible design to construct DMATS and (2) test the precision and accuracy of DMATS.

## Methods

### System design

Equipment used in the construction of DMATS was an electric drill with a hole cutting attachment, circular saw, and measuring tape. The design for DMATS includes a detachable base, antenna mast, four-element Yagi directional antenna, and electronic compass. All wood used in the assembly of DMATS was green treated and construction grade. Green treated wood was used to prevent rotting as a result of the base being exposed to various weather conditions. Prior to assembly, two coats of water sealant were applied according to the manufactures instructions for the application of multiple coats and all screws affixed to the detachable base were sealed with silicone after insertion. All measurements provided for the construction of DMATS include the metric values followed by the standard American equivalent (SAE) of inches (in) and feet (ft).

**Figure 1:**
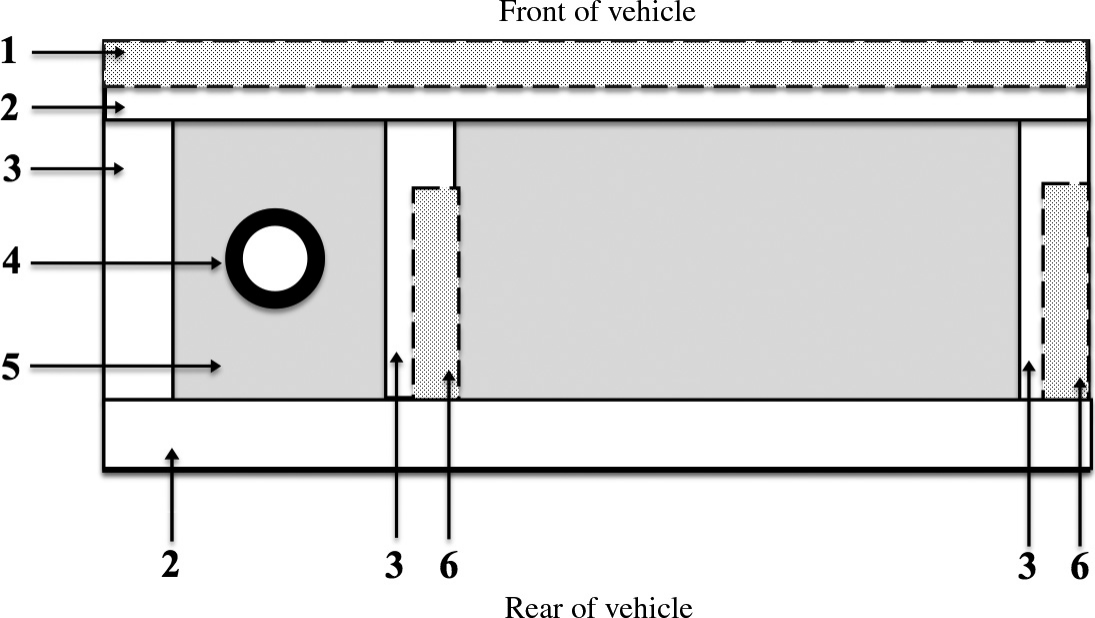
Schematic design for the base of the detachable mobile and adjustable telemetry system (DMATS). The description of each component is followed by the dimensions: (1) stud, stabilizer (5.08 × 7.62 cm × 1.14 m; SAE: 2 × 3 in × 3¾ ft), (2) stud, reinforcing (5.08 × 10.16 cm × 1.83 m; SAE: 2 × 4 in × 6 ft), (3) stud, reinforcing (5.08 × 10.16 cm × 1.83 m; SAE: 2 × 4 in × 6 ft), (4) flange (3.81 cm; SAE: 1½ in), (5) green treated plywood (45.72 × 1.83 m × 2.54 cm; SAE: 18 in × 6 ft × 1 in), (6) stud, stabilizer (5.08 × 7.62 × 45.72 cm; SAE: 2 × 3 × 18 in). Dotted lines indicate additional support pieces added to the bottom of the base component is followed by the dimensions: (1) stud, stabilizer (7.62 cm × 1.14 m × 5.08 cm), (2) stud, reinforcing (7.62 cm ×1.83 m × 5.08 cm), (3) stud, reinforcing (7.62 × 45.72 × 5.08 cm), (4) flange (3.81 cm), (5) green treated plywood (45.72 cm × 1.83 m × 2.54 cm), (6) stud, stabilizer (7.62 × 45.72 × 5.08 cm).

Construction for the base consists of a 45.72 cm × 1.83 m× 2.54 cm (SAE: 18 in × 6 ft × 1 in) section of green treated plywood reinforced by a framework of 5.08 × 10.16 cm (SAE: 2 × 4 in) studs. Two 1.83 m (SAE: 6 ft) long studs were placed lengthwise, parallel to each other on the outer edges of the base even with the outer edges of each stud, and two 30.48 cm (SAE: 12 in) long studs placed widthwise (forming a box by connecting the two perpendicular studs), parallel to each other on opposite ends of the base (Fig. 1). These studs were added on the outer portion of the plywood for additional support and stability. An additional 30.48 cm (SAE: 12 in) stud was placed 45.72 cm (SAE: 18 in) from one end of the stud across the entire width of the platform. The positioning of this stud is important as it creates 45.72 × 45.72 cm (SAE: 18 × 18 in) reinforced area for the antenna mast. Using an electric drill, each stud was affixed to the base with 7.62 cm (SAE: 3 in) wood screws.

A 3.81 cm (SAE: 1½ in) diameter hole was centered and cut into the 45.72 × 45.72 cm (SAE: 18 × 18 in) reinforced area using a hole cutter. Two 3.81 cm (SAE: 1½ in) flanges were centered over the hole, one on the top and bottom of the base, then secured with bolts and locking nuts. This served as the entry point for the 2.54 cm × 3.05 m (SAE: 1 in × 10 ft) aluminum pole used as the antenna mast. The base was covered in 1.27 cm (SAE: ½ in) thick padding, then covered with weatherproof outdoor carpeting to prevent damage to the vehicles painted surface. The padding and carpeting were secured to the base using wood screws inserted to a depth below the surface of the base and outdoor carpet adhesive.

**Figure 2:**
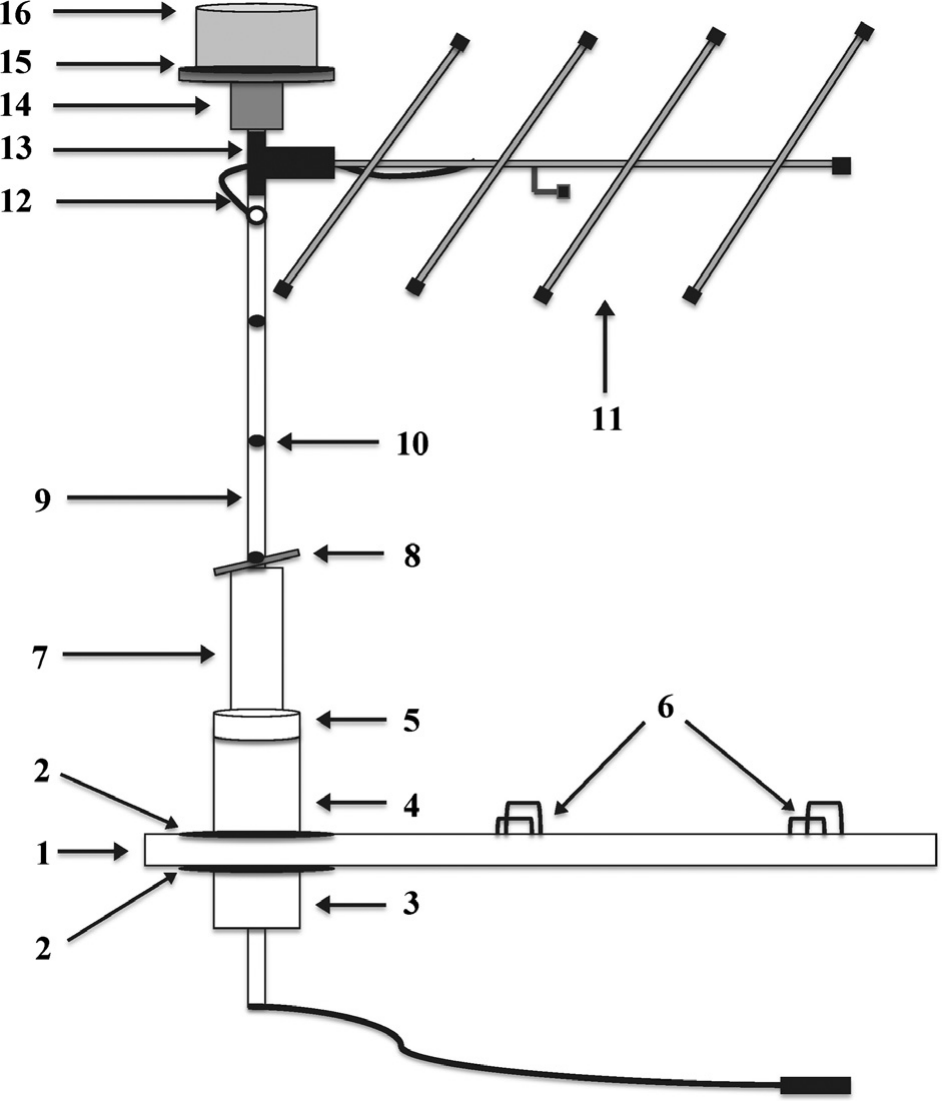
Schematic design of the completed base, support shaft assembly for the mast, antenna mast, and directional antenna for detachable mobile and adjustable telemetry system (DMATS). The description of each component is followed by the dimensions: (1) base (45.72 cm × 1.83 m × 12.70 cm; SAE: 18 in × 6 ft × 5 in), (2) flange (3.81 cm; SAE: 1½ in), (3) aluminum nipple (3.81× 10.16 cm; SAE: 1½ in), (4) aluminum nipple (3.81 × 30.48 cm; SAE: 1½ × 12 in), (5) compression coupling (2.54 × 3.81 cm; SAE: 1 × 1½ in), (6) U-bolts (2.54 cm; SAE: 1 in), (7) polyvinyl chloride (PVC) pipe (3.18 × 45.72 cm; SAE: 1¼ × 18 in), (8) cotter pin, (9) antenna mast (2.54 cm × 3.05 m; SAE: 1 in × 10 ft), (10) height adjustment holes (6.4 mm; SAE: ¼ in), (11) directional antenna, (12) antenna cable (7.62 m; SAE: 25 ft) (13) antenna mounting bracket, (14) spindle, (15) wooden disc spacer (17.78 × 1.91 cm; SAE: 7 × ¾ in), (16) electronic compass.

A 3.81 cm (SAE: 1½ in) compression coupling was attached to a 3.81 × 30.48 cm (SAE: 1½ × 12 in) aluminum nipple. The nipple was placed on the flange at the top of the base (Fig. 2). A 3.18 × 45.72 cm (SAE: 1¼ × 18 in) section of polyvinyl chloride piping was inserted into the free end of the compression coupling to complete the support-shaft assembly for the antenna mast. As an additional measure of support for the antenna mast, a 3.81 × 10.16 cm (SAE: 1½ × 4 in) nipple was also placed on the flange located on the bottom of the base. We then sealed all nonrotating connection points with silicone to prevent water from entering those connection points.

A U-bolt was placed at each corner of the platform using a 2.54 cm (SAE: 1 in) bracket with locking nuts. This also served as the connection points for the ratcheting tie downs (Fig. 2). It may be necessary to vary the exact location of the U-bolts based on the size of the vehicle. It is important to note that this must be considered prior to construction of the base, as this will drastically affect the weight distribution. The base was secured to the top of the vehicle by fastening one end of a tie down to the base and the other end to chassis of the vehicle and ratchet until taut. This was repeated for each U-bolt. It must also be noted that no U-bolts were placed on the driver's side of the vehicle, as this would hinder the opening of the driver's door. To add stability and balance to the base once on the vehicle, additional 5.08 × 7.62 cm (SAE: 2 × 3 in) studs were placed at the front of the base to compensate for the curvature in the roof of the vehicle (Fig. 1).

Four 6.4 mm (SAE: ¼ in) holes, spaced 30.48 cm (SAE: 12 in) apart, were drilled through the mast to allow for the adjustment of mast height. A steel cotter pin was placed through the 6.4 mm (SAE: ¼ in) hole in the mast and lowered to rest on the top of the support shaft assembly. This prevents the further lowering of the mast while maintaining the ability to rotate a full 360°. A four-element Yagi antenna (Advanced Telemetry Systems, Inc.; Isanti, MN) was attached to the pole using the mounting hardware supplied by Advanced Telemetry Systems (ATS). A 1.59 cm (SAE: 5/8 in) diameter hole was drilled into one side of the mast and a 7.62 m (SAE: 25 ft) coaxial antenna cable was threaded through the center of the mast and down into the cabin of the vehicle where it was attached to an ATS Challenger telemetry receiver (model number R2000).

**Figure 3:**
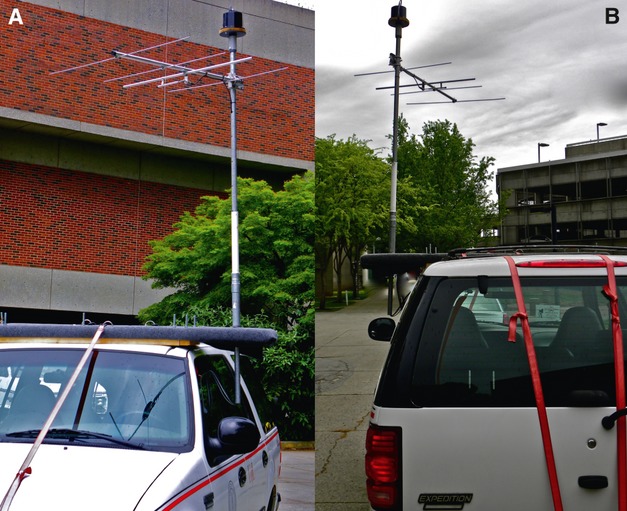
Front (A) and rear (B) view of the detachable mobile and adjustable telemetry system (DMATS) mounted on a university sports utility vehicle.

**Figure 4:**
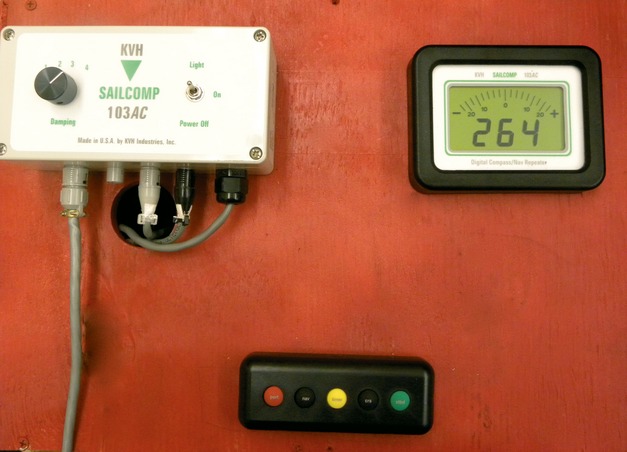
Junction box (top left) in which a Sailcomp 103 AC (KVH Industries, Inc.; Middletown, RI) electronic compass (not pictured) was connected. Also shown is the digital compass display (top right) and remote keypad (bottom).

A Sailcomp 103 AC (KVH Industries, Inc.; Middletown, RI) electronic compass was used to increase the accuracy of azimuth (bearings) readings (Brinkman et al. 2002; Cox et al. 2002). The compass was comprised of an Autocomp 1000 remote heading sensor, remote digital display, remote keypad, and junction box. We affixed the heading sensor to a thin, <0.5 cm (SAE: 3/16 in) thick, film of rubber then secured the sensor to a 17.78 cm (SAE: 7 in; diameter) and 1.91 cm (SAE: ¾ in; thickness) wooden spacer. The manufacturer of the compass recommended the compass be placed a minimum distance of 30.5 cm (SAE: 12 in) from magnetic materials; therefore, the compass was attached to a spindle and affixed to the top of the antenna mast 45 cm (SAE: 18 in) above the antenna (Fig. 2). A small notch, ∼3.81 cm (SAE: 1½ in), large enough for the display and power cables of the compass, was cut into the wooden spacer to allow the cables from the electronic compass to feed through to the center of the mast. We then fed the cables from the electronic compass into the hole previously created for the antenna cable, out the opposite end of the mast, and into the cabin of the vehicle (Fig. 3). Once inside the vehicle, both cables were connected to the junction box, which was connected to a 12-volt rechargeable battery (Fig. 4). The electronic compass and antenna were synchronized by aligning the main tine of the antenna with the directional arrow on the remote heading sensor of the electronic compass. We then calibrated the electronic compass by following the manufacturer's directions.

### Study location

**Figure 5:**
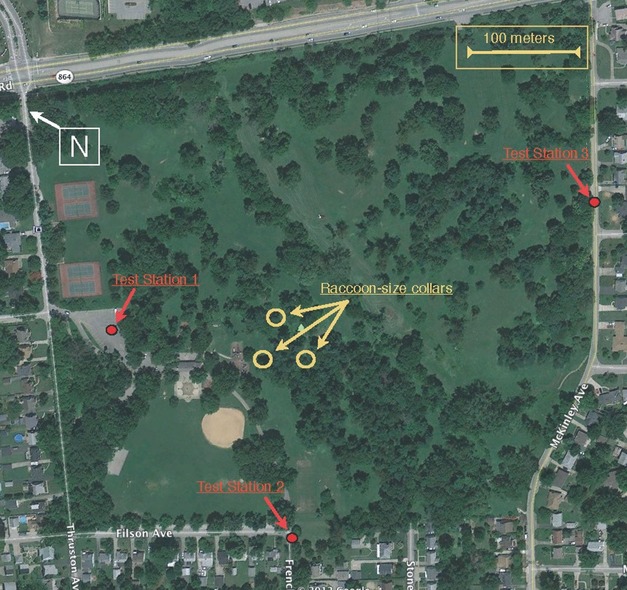
George Rogers Clark Park located in Louisville, Kentucky where the accuracy and precision of the Detachable Mobile Adjustable Telemetry system was tested. Figure includes the locations of the testing stations and radio collars used in the system evaluation.

We tested the accuracy and precision of the DMATS in George Rogers Clark Park in Louisville, Kentucky (Fig. 5). The park is 18.8 ha in size and surrounded on three sides by single-family homes and a high traffic four-lane road on the fourth side. The park varies greatly in elevation, 143–155 m, and vegetation density. Steep slopes rise from a streambed that runs diagonally across the park and regularly mown fields are interspersed with large trees and dense clusters of shrubs. This area was selected as a test location because it is representative of neighborhood parks and adjacent landscapes (Parker and Nilon 2012).

### System evaluation

The accuracy of DMATS was evaluated by measuring the error distances (the distance between the estimated location and actual location of the radiocollar) and the precision (the ability to repeat the same measure with statistically similar results) of DMATS by evaluating the size of error ellipses and the associated standard deviation. As a guide for acceptable results, we compared the level of accuracy and precision of DMATS to the level of accuracy and precision of a handheld system consisting of an identical receiver and a three-element Yagi antenna. This configuration for the handheld system was selected, as it is the most commonly used configuration for handheld telemetry systems (Kenward 2001). Additionally, the approach of comparing novel mobile configurations to handheld configurations is common and has been used in previous studies by Brinkman et al. (2002), Cox et al. (2002), and Fuller et al. (2005); therefore, we deemed this methodology sufficient to evaluate the accuracy and precision DMATS.

In October 2012, four technicians (testers) collected azimuths from raccoon sized (46 g) radiocollars broadcasting at 149.000–149.999 MHz with a pulse rate of 40 ppm (Advanced Telemetry Systems; Isanti, MN; model number M1920). Three testers used DMATS and one the hand-held system. All of the DMATS testers were previously trained in telemetry and recorded azimuth readings weekly for a minimum of 1 year prior to testing. The tester using the handheld system had >16 years experience performing telemetry.

We placed three radiocollars 50 m apart and 1.2 m above the ground by attaching each radiocollar to a wooden stake using twined string. The broadcasting frequency for each collar was noted and coordinates of each radiocollar recorded using a Magellan Triton 2000 GPS unit (Santa Clara, CA). We then established three testing stations, ∼200–280 m from the locations of the radiocollars, and recorded the coordinates of each testing station with the Magellan Triton 2000. A number was assigned to each collar (1–3) and testing station (1–3). We entered the numbers of the collars and testing stations into a random numbers generator to determine the sampling sequence for every trial for each tester.

Prior to the trial, no tester was aware of the collar to be located or the order of the testing stations from which the azimuth would be taken. All azimuths taken by testers using DMATS were conducted from the lowest height of the antenna, 3 m – as this would be the closest height to that of the handheld system. Additionally, the user of the handheld system held the yagi antennae to the highest point over the head, 2.5 m. Each trial consisted of three azimuths taken for a radiocollar, which was predetermined before the initiation of the trial, from each of the testing stations.

Error ellipses, the area within which a location estimated from two or more bearings is likely to occur (Saltz 1994; Kenward 2001), were determined by entering bearings from the electronic compass into Locate III, version 3.33 (Nams 2006) telemetry software which was accessed through a Hewlett-Packard iPAQ personal digital assistant (PDA). Error distance, the difference between the estimated location and the true location of the radio collar, was calculated for each trial. We used a one-way analysis of variance (ANOVA) with a Scheffe post-hoc test to examine differences in error ellipses and error distances between DMATS and the handheld system by using the statistical package SPSS (IBM Corporation, Armonk, NY).

## Results

### System design

The construction of DMATS required a total of 15 labor hours. This consisted of construction of the base (6 h), antenna mast and support (4 h), mounting of the antenna (1.5 h), and affixing the compass to the antenna mast (3.5 h). The total cost for supplies to assemble DMATS was $1059 USD, with the largest of those cost being the Sailcomp 103AC electronic compass, $695 USD (Table 1).

**Table 1:** Summary of cost for the materials to build the detachable mobile and adjustable telemetry system

	Quantity needed	Item price	Item total
Base			
Plywood	1	$40.00	$40.00
Studs	2	$15.00	$30.00
Flanges	2	$6.00	$12.00
Padding	1	$10.00	$10.00
Outdoor carpeting	1	$11.00	$11.00
Total			$103
Antenna and mast			
Antenna	1	$125.00	$125.00
Aluminum pole	1	$12.00	$12.00
Aluminum nipple	2	$6.00	$12.00
Electronic compass	1	$695.00	$695.00
Compression coupling	1	$3.00	$3.00
PVC piping	1	$8.00	$8.00
Total			$855.00
Hardware			
Screws	1	$6.00	$6.00
Bolts	1	$4.00	$4.00
Nuts	1	$4.00	$4.00
U-bolt	4	$1.00	$4.00
Steel cotter pin	1	$2.00	$2.00
Total			$20.00
Miscellaneous			
Water sealant	1	$10.00	$10.00
Silicone	1	$6.00	$6.00
Carpet adhesive	1	$10.00	$10.00
Wooden spacer	1	$4.00	$4.00
Rubber film	1	$2.00	$2.00
12-volt battery	1	$4.00	$24.00
Ratcheting tie downs	1	$25.00	$25.00
Total			$81.00
Total for DMATS			$1059.00

Prices are given in 2012 US dollar.

### System evaluation

All testers conducted 12 trials after three trails to acclimate to the topographic and environmental conditions of the test location (Table 2). Mean error ellipses ranged from 0.22 ± 0.34 ha to 5.85 ± 16.07 ha for DMATS testers. The overall mean error ellipses was 1.95 ± 1.22 ha. The tester using the handheld telemetry system had the smallest error ellipses, 0.06 ± 0.02 ha, while tester three had the largest, 5.85 ± 16.07 ha. Results of the one-way ANOVA with a Scheffe post hoc test for error ellipses yielded no significant differences between DMATS testers and the handheld telemetry system (*P* = 0.292).

**Table 2:** Location estimates for testers of the Detachable Mobile Adjustable Telemetry System (DMATS)

Tester	*N*	Error ell (ha)	Error dist (m)
1	12	0.22 ± 0.34	58.15 ±5.83
2	12	1.68 ± 2.92	81.50 ± 57.28
3	12	5.85 ± 16.07	65.18 ± 30.51
4	12	0.06 ± 0.02	20.02 ± 11.12
Total	48	1.96 ± 8.33	56.21 ± 39.27

*N* represents the number of trials conducted for each tester. Mean error ell is the mean error ellipse (the average polygon area containing the true radio collar location) followed by the standard deviation, both given in hectares. Mean error dist is the error distances (the difference between the estimated location and the true location of the radio collar) in meters followed by the standard deviation.

**Figure 6:**
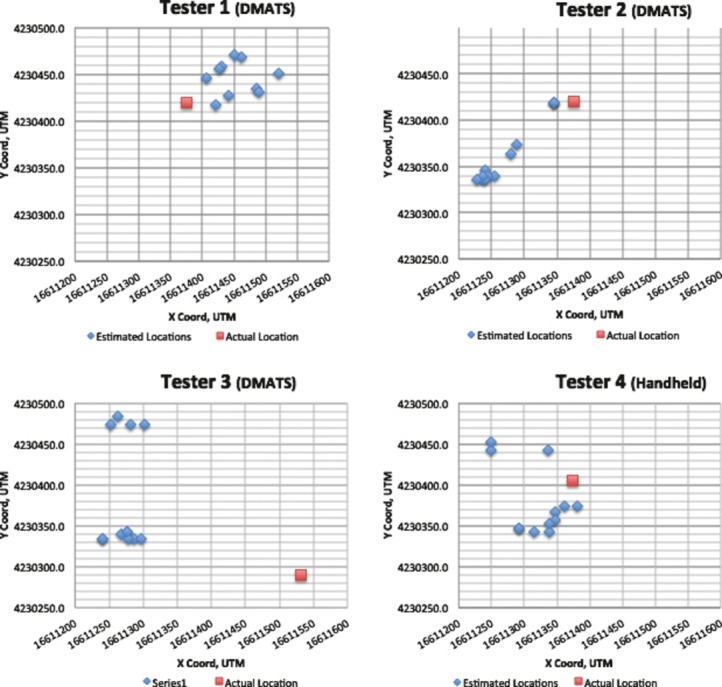
Scatterplot of true location (red) versus estimated locations (blue) of radiocollars used in test trials. *X* axis is *x*-coordinate and y axis is *y*-coordinate. All coordinates are given in Universal Transverse Mercator (UTM) coordinate system. All testers completed 12 trials (*n* = 12). Testers 1–3 used DMATS for each trial and tester four used a handheld telemetry system.

The average error distance for all DMATS testers was 56.21 ± 39.27 m (Table 2). Error distances ranged from 20.02 ± 11.12 m for the handheld system to 81.50 ± 57.28 m for tester two using DMATS. The mean error distance for DMATS testers was 68.28 ± 31.21. Scatterplots of the true versus estimated locations of the radiocollars yielded high levels of groupings in close proximity to the true locations (Fig. 6). Comparisons between individual testers yielded significant differences between testers two and four (*P* = 0.00) and three and four (*P* = 0.02).

## Discussion

### System design

The digital mobile and adjustable telemetry system being introduced may benefit researchers lacking a dedicated vehicle in which permanent alterations can be made. The construction of DMATS is simple and can be accomplished in a relatively short period of time given the necessary materials and tools are available. The cost for materials is small. However, the electronic compass, the most costly portion of the equipment, can be substituted with a compass rose that has some type of pointed object that has been aligned with the main tine of the antenna that can be used to indicate the bearing on the compass rose. If this is not an option, the user can utilize a handheld compass by exiting the vehicle and taking a reading by aligning the handheld compass bearing with the main tine of the antenna. Both methods, which will lower the cost of DMATS, also come with the potential for increasing the probability of increased human error in acquiring a bearing when compared to that of an electronic compass. Kenward (2001) described methods to construct a traditional mobile system, which requires a permanent hole to be placed in the roof of the vehicle's cabin, and estimated the cost to be $2100–$4200 USD (these amounts have been adjusted to 2012 values). Far more expensive than the $1059 USD needed for the construction of DMATS.

### System evaluation

The accuracy and precision of DMATS were important factors when designing this system. We defined accuracy as the ability of the device to correctly identify the true location of the telemetry collar and precision as the ability of DMATS to identify the true location of the telemetry collar repeatedly. We assessed the accuracy of DMATS by the size of error ellipses and the bias between the estimated location of the collar and the true coordinates of the radio collar. Additionally, we assessed precision by examining the standard deviations associated with the error ellipses and the distribution of estimated locations.

The absence of statistical difference in the size of error ellipses between the handheld system and DMATS indicated that the precision of DMATS proved to be equivalent to that of the handheld telemetry system. However, even though there was variation in the size of error ellipses between users the standard deviations, which is the measure of precision for telemetry (Saltz 1994; Kenward 2001; Fuller et al. 2005), remained relatively small for each tester (Table 2). Demonstrating the precision, or repeatability, of the system. Furthermore, even with the variation in the sizes of error ellipses, the areas within the error ellipses overlapped. It is those areas of overlap that contained the true location of the test collar.

We believe the primary cause for variation in error ellipses was due to the differing levels of experience between the testers and topographic features of the area used for testing the system. In difficult terrain, detecting the direction of the strongest signal can be challenging for the most experienced technician (Kenward 2001). However, when combining the topography with the presence of electrical power lines, such as those located within and surrounding the test location, and more specifically, those near testing stations, signal deflection may have increased substantially. Therefore, making the detection of the actual location of the collar difficult to distinguish—especially by a less experienced user.

We believe some features of DMATS may help minimize mistakes by the less experienced user. In most cases, the more experienced user will be knowledgeable of methods to reduce some biases associated with telemetry, however, the ability to adjust antenna height and the use of an electronic compass may compensate for the lack of experience. The ability to adjust the antennae is critical to getting the strongest signal reception (Kenward 2001; Fuller et al. 2005). In challenging habitats, especially those with increased signal deflection, less experienced users can become frustrated trying to identify the bearing of the signal. Having the ability to not just move the antennae horizontally but also vertically will greatly increase the ability of the user to detect the true signal bearing.

Mountainous, afforested, and urban areas can prove to be quite challenging for telemetry studies. Kenward (2001) noted difficulties in identifying a bearing because of signal deflection in areas with considerable amount of tree cover. However, while testing DMATS in an urban setting, we also took note of the sizeable amount of signal deflection. Given the inherent characteristics of urban settings, increased electrical wiring and high concentrations of impervious surfaces, the occurrence of signal deflection may be a continuous. As such, this will almost certainly create difficulties in determining the true bearing of transmitting devices. However, we believe, in addition to the adjustable antenna height, the use of the electronic compass, may make the task of achieving a true signal bearing more attainable for less experienced users. While testing DMATS, the electronic compass allowed the user to focus on correctly identifying the direction of the strongest signal reception without the distraction of determining the bearing using a compass rose or a prismatic compass.

Mobile telemetry systems possess an advantage over handheld telemetry systems by allowing the user to move more rapidly between sampling areas. Additionally, the use of a vehicle removes the constraints of the weight and difficulty associated with carrying added equipment or a larger antenna. Our mobile telemetry system provides the traditional advantage over handheld telemetry while also providing adaptability not found with other vehicle-mounted systems. The DMATS system allows the user to cover multiple terrain types by simply moving the system to an appropriate vehicle. Users who may otherwise track animals with a handheld system due to an inability to modify a vehicle or multiple vehicles can utilize DMATS to decrease search time and increase search range.

As an added benefit, although not tested empirically, we believe the use of DMATS increased the speed in which readings can be taken when compared to that of a handheld system. We make this assumption because DMATS allows the operator to take a bearing from the driver's seat of the vehicle, eliminating the time used to park and exit the vehicle. This can be important when performing telemetry in urban settings or other areas where the necessary locations to take readings may be unsafe for vehicles to be stopped for prolonged instances. Additionally, the use of the digital display of the electronic compass also increased efficiency by reducing the amount of time that would be needed to determine a compass bearing. This may be of greater importance when tracking highly mobile species and minimizing time between readings is important.

## Conclusions

Having the proper radiotelemetry equipment is a critical step in gathering accurate wildlife and habitat association data applicable to behavior or population biology studies. If the system is adequately assembled and tuned, then the ability to collect accurate data may rest primarily on the limitations of the user (Fuller et al. 2005). These limitations can be a lack of understanding the capabilities and limits of the equipment, challenges from the environment in which the equipment is being used, and the intrinsic difficulties presented by the species of the focal animal. The accuracy and precision of DMATS proved to be equivalent to that of the traditional handheld system. As a result of this, we believe this system can be of great utility to any researcher regardless of habitat type.
